# Presentation format influences the strength of causal illusions

**DOI:** 10.3758/s13421-025-01714-z

**Published:** 2025-04-29

**Authors:** Ainoa Barreiro, Anadaniela Del Carpio, Javier Rodríguez-Ferreiro, Itxaso Barberia

**Affiliations:** Grup de Recerca en Cognició i Llenguatge (GRECIL), Departament de Cognició, Desenvolupament i Psicologia de L’Educació, Secció de Processos Cognitius, Institut de Neurociències (UBneuro), Facultat de Psicologia, Universitat de Barcelona (UB), Passeig de La Vall d’Hebron, 171, 08035 Barcelona, Spain

**Keywords:** Causal illusion, Contingency detection, Frequency tree, Icons, Contingency table

## Abstract

Causal illusions refer to the erroneous perception of causal connections between noncontingent variables. Previous research has demonstrated that the format in which contingency information is displayed can impact causal judgments. On this basis, we examined the effect of graphical displays on the strength of causal illusions and reasoning strategies across three experiments. Study 1 revealed that frequency trees and contingency tables involving icons lead to weaker causal illusions than trial-by-trial presentations or contingency tables with numbers. An assessment of the participants’ open responses in Study 2 indicated that stronger causal illusions were associated with reports of less sophisticated reasoning strategies. In Study 3, we directly compared frequency trees and contingency table visualizations. In addition to corroborating previous observations, we found that advanced strategies were more likely when the information was presented in frequency trees. Overall, our findings suggest that the efficacy of frequency trees in reducing causal illusions may be due to their ability to make sophisticated strategies more accessible.

## Introduction

Causal reasoning is essential to navigate and understand the world that we live in and can have a direct impact on how we act upon the environment. For instance, if one tends to experience stress relief after exercising, one will be more likely to exercise regularly during stressful periods. Research suggests that people can be fairly accurate at assessing causality (Chatlosh et al., 1985; Wasserman et al., 1983). Nevertheless, some reports suggest that sometimes people experience causal illusions (see Matute et al., 2015, for a review). In other words, they perceive a causal connection between two variables that are actually noncontingent (Matute et al., 2019). Beyond being a laboratory phenomenon, this cognitive bias might influence our daily lives, as shown by the association between the proneness to develop causal illusions and poorer fake-news discriminability (Saltor et al., 2023), as well as endorsement of pseudoscientific (Torres et al., 2020, 2022) and paranormal beliefs (Blanco et al., 2015; Griffiths et al., 2018).

**Fig. 1:**
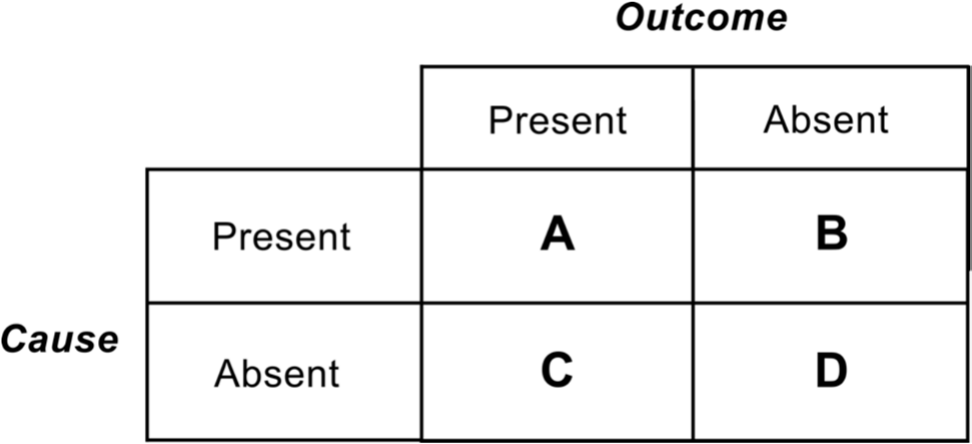
A contingency table illustrating the data needed to derive conclusions about cause–effect scenarios. *Note*. A, the frequency of cases in which the potential cause and outcome are present; B, the frequency of cases in which the potential cause is present, but the outcome is not; C, the frequency of cases in which the potential cause is absent, and the outcome is present; D, the frequency of cases in which the potential cause and the outcome are both absent

Causal illusions have been thoroughly studied in previous research on causal learning (e.g., Barberia et al., 2019; Blanco et al., 2013; García-Arch et al., 2024). Frequently, these studies are based on contingency learning tasks, in which participants must indicate to what extent they believe there is a causal relationship between a putative cause (C) and an outcome (O). To be able to judge the relationship between these two variables, in a series of trials, participants are given information about whether the potential cause (e.g., a fictitious drug) is present or not, followed by information about the presence or not of the outcome (e.g., recovery from a health condition). In essence, the participants are given instances of the four possible combinations of the two variables, which can be summarized in a 2 × 2 contingency table: (A) cases in which the potential cause and outcome are present; (B) cases in which the potential cause is present, but the outcome is not; (C) cases in which the potential cause is absent, and the outcome is present; (D) cases in which the potential cause and the outcome are both absent (see Fig. 1).

Thus, in these experiments, trials corresponding to each cell (*A*, *B*, *C*, and *D*) are sequentially presented in random order. After presenting the trials, participants must rate on a numerical scale the extent to which the potential cause impacted the outcome. Often, the scale rating ranges from 0 to 100, with higher ratings indicating a stronger causal influence of the putative cause over the outcome.

A mathematical approach for assessing the contingency's strength and direction is applying the ΔP rule (delta rule; Jenkins & Ward, 1965). The ΔP index is calculated by computing the difference between the probability of the outcome given the presence of the cause, P(Outcome|Cause), and the probability of that outcome given the absence of the cause, P(Outcome|¬Cause):

1 $$\Delta P = P\left(Outcome|Cause\right)- P\left(Outcome|\neg Cause\right)$$

Based on the information in the cells presented in Fig. 1, the ΔP rule can easily be computed with the following formula:

2 $$\Delta P = \frac{A}{A + B}-\frac{C}{C + D}$$

The index ranges from − 1 to 1, with positive numbers indicating that the presence of the cause enhances the probability of the appearance of the outcome. In contrast, negative values imply that the presence of the cause diminishes the likelihood of the outcome’s appearance. Moreover, when the ΔP takes the value of 0, the cause has no impact on the probability of the outcome. Causal illusions arise when ΔP is 0, but people develop the impression that the two variables are causally connected.

Note that the ΔP rule has been proposed as a normative method to assess causal strength (although see Cheng, 1997, for an alternative proposal) and, in fact, studies have found that people’s judgments can align with this rule (Chatlosh et al., 1985; Shanks & Dickinson, 1987; Wasserman et al., 1983, 1993). Nevertheless, there have also been studies suggesting that judgments approximate to the results of less sophisticated strategies (Shaklee & Tucker, 1980; Ward & Jenkins, 1965). Traditionally, some of the rules that have been proposed are the Cell A, A–B, and the sum of the diagonals’ strategy (for other approaches, see Perales et al., 2017; see also Perales & Shanks, 2007). The Cell A strategy (Nisbett & Ross, 1980; Smedslund, 1963) is the simplest heuristic and consists of assessing contingencies based solely on the information in Cell A (see Fig. 1). In other words, the higher the frequency of co-occurrence of the candidate cause and the outcome, the higher the causal judgment. On the other hand, in the A–B strategy, the assessment of contingency is based on comparing the frequencies in Cells A and B (Inhelder & Piaget, 1958; Shaklee & Mims, 1982). Lastly, the sum of the diagonals’ strategy (Inhelder & Piaget, 1958; Shaklee & Tucker, 1980) involves comparing the number of confirming cases (Cell A + Cell D) with the number of disconfirming cases (Cell B + Cell C). This last method is much more sophisticated than the Cell A and A–B strategies, but it can also result in assessments that are far from the normative value provided by ΔP.

As mentioned, studies on causal learning typically present information sequentially (Barberia et al., 2019; Blanco et al., 2013; Jenkins & Ward, 1965). This is probably associated with the fact that sequential procedures closely simulate how causal relationships are often learnt in natural environments. For example, we might learn about the effectiveness of a remedy (the candidate cause) for producing relief (the outcome) from a health condition by successive occasions in which we are feeling sick and decide to take the remedy or not. Nonetheless, in our contemporary society, summarized visualizations are frequently used to communicate ideas and findings regarding causal connections. For instance, we might encounter a graph summarizing the results of a clinical study that tested the effectiveness of a drug; or a table indicating the number or rate of treated and placebo patients that experienced a positive outcome after an intervention. Given the proliferation and increasing access to this type of condensed information, it is worth questioning whether summarized displays are less prone to producing causal illusions and which specific summarized formats work better.

In this regard, some studies in causal reasoning have tried to compare performance in trial-by-trial procedures and contingency tables. More precisely, Ward and Jenkins (1965) assessed whether showing the information trial-by-trial, in a contingency table, or both trial-by-trial plus in a contingency table would have an impact on the strategy used to make the causal judgments. The results showed that, when presenting the data in a contingency table, participants were more likely to use the ΔP rule, thus making more normative judgments. Similarly, Arkes and Harkness (1983, Experiments 4 and 5) found that when assessing contingency, individuals were more inaccurate when the information was presented trial-by-trial than when it was presented in a table. Interestingly, when using contingency tables, the most predominant heuristic participants used to solve the problem was the sum of the diagonals, followed by conditional probability comparison (i.e., the ΔP rule) and Cell A. In contrast, in the trial-by-trial display, the most frequent strategy was Cell A, followed by A–B, the sum of the diagonals, and the ΔP rule. Along the same lines, Kao and Wasserman (1993) studied participants’ judgments on noncontingent problems presented either sequentially or in a table. The results showed that causal ratings were more inaccurate (i.e., deviated more from zero) for the trial-by-trial than for the contingency table display. On top of that, they also examined the strategies participants utilized, which showed that the use of the ΔP rule was more prevalent when employing contingency tables.

**Fig. 2:**
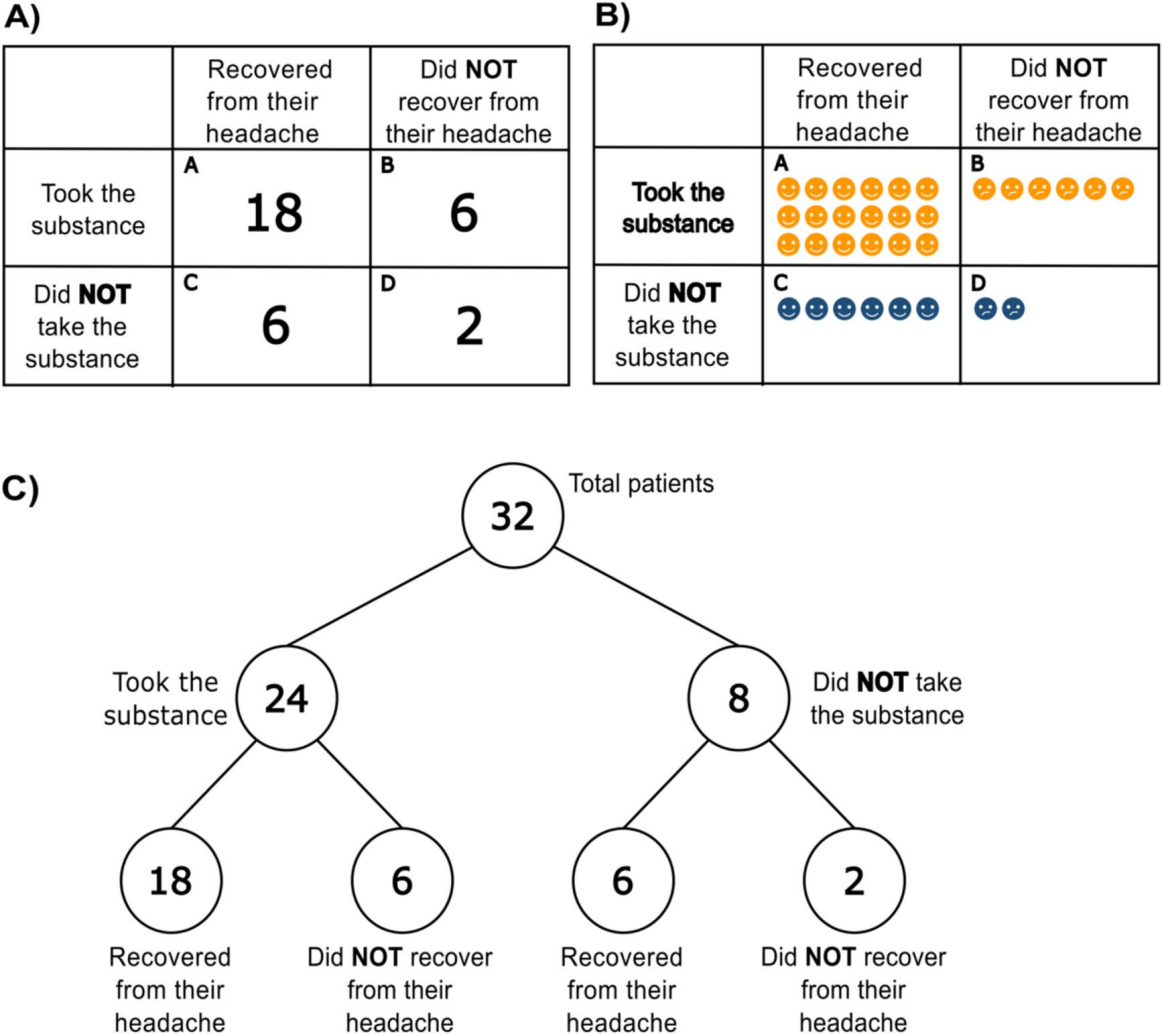
Information visualization formats: **A)** contingency table with numbers, **B)** contingency table with icons, and **C) **frequency tree. (Color figure online)

These prior findings on causal reasoning point out that summarized information in contingency tables may enhance the accuracy of causal judgments. Nevertheless, there are other formats in which data can be summarized, such as contingency tables with icons (see Fig. 2B), frequency trees (see Fig. 2C), bar graphs, or mosaic plots. Although the literature is scarce in this regard, some studies have tried to compare the efficacy of different summarized formats on causal inferences.

For instance, Cooper and Vallée-Tourangeau (2021) observed that participants tended to provide more accurate judgments when the information was presented in a bar graph rather than a contingency table. Furthermore, these displays have also been investigated as possible instruments for debiasing confirmation bias in causal judgments. Confirmation bias is defined as the tendency to seek and interpret evidence so that it confirms our preexisting beliefs (Nickerson, 1998). Xiong et al. (2024) showed that when using contingency tables, the confirmation bias was reduced, and, therefore, people were more likely to respond accurately compared with when information was displayed in a bar graph or a bar chart.

Besides contingency tables and bar graphs, some authors have studied the use of frequency trees. Vallée-Tourangeau et al. (2008) asked participants to solve six contingency problems, where ΔP was zero for three of them, and 0.50 for the rest. Contingency information could be presented in a frequency tree, a contingency table with icons, or a written format. They found that participants who visualized information on a frequency tree were more prone to discriminate between a positive and a zero contingency than those who were presented with a contingency table or a written format. Although not directly related to contingency learning, it is worth noting that frequency trees have also been used in numerous studies on Bayesian reasoning (Böcherer-Linder et al., 2017; Eichler et al., 2020; Yamagishi, 2003). Indeed, research shows higher performance on Bayesian problems when information is visualized in a frequency tree rather than in written form with probabilities or natural frequencies (Binder et al., 2015, 2018, 2021; Friederichs et al., 2014; Yamagishi, 2003).

In contrast, several studies have used icons to represent contingency information (Liljeholm & Cheng, 2009; Stephan & Waldmann, 2018). However, to our knowledge, their potential use to enhance reasoning has only been explored by Vallée-Tourangeau et al. (2008), who presented the icons in a contingency table. Their results were not very robust regarding their potential generalizability. In fact, these authors found that icons only helped to differentiate between a positive and null contingency when the P(Outcome|Cause) was 0.75. In contrast, when the P(Outcome|Cause) was 0.50, the participants could not discriminate between them. Although the findings on the use of icons in causal reasoning are scarce, their usefulness has also been reported with regard to Bayesian reasoning (Bancilhon et al., 2023; Tubau et al., 2019, 2023; Walker et al., 2023). Specifically, for solving Bayesian problems, icons seem to be more useful than Venn circles, roulette wheels, and unit squares visualizations (Cui et al., 2023). On top of that, they also help reduce the denominator neglect bias, which is the tendency to focus on the numerator of ratios while ignoring the denominator (Garcia-Retamero et al., 2010; Okan et al., 2012).

In summary, previous results suggest that the layout of the information impacts the way people reason and that not all the summarized formats are equally effective at facilitating normative judgments. Thus, we believe that determining which format allows for more accurate causal estimations can be a powerful tool to help people make better causal judgments and reduce their causal illusions.

Studies comparing trial-by-trial and summarized formats have mainly focused on contingency tables with numbers (Arkes & Harkness, 1983; Kao & Wasserman, 1993), and occasionally in written formats (Mandel & Vartanian, 2009). Concurrently, research on graphical display has tested a more varied range of formats. However, to our knowledge, there has been no attempt to bridge these two research trends and provide a more unified perspective on the role of presentation format in the context of causal illusions. For this reason, this research sought to investigate the impact of visualizations on the assessment of noncontingent information. Our primary focus was identifying displays that may aid in diminishing causal illusions and comprehending the contributing factors to this asset. In Study 1, given that causal illusions have been mainly studied through trial-by-trial procedures, we sought to investigate whether different summarized formats could become a viable strategy to reduce these illusions. We compared four displays (i.e., trial-by-trial, contingency tables employing numbers, contingency tables involving icons, and frequency trees) in a between-subjects design. We expected that the format in which the information was presented in the contingency problem would have an impact on the causal illusion generated. The causal ratings would be closer to zero (i.e., more accurate) when depicting noncontingent information summarized in a contingency table or tree format, compared with exhibiting the information sequentially (Arkes & Harkness, 1983; Kao & Wasserman, 1993; Ward & Jenkins, 1965). In addition, we also hypothesized that the type of graphical display used when presenting the summarized information would affect causal judgments. Showing the information in the form of a frequency tree would help mitigate the errors in causal judgment in comparison to when displaying information in a contingency table, either with icons or numbers (Bancilhon et al., 2023; Binder et al., 2021; Vallée-Tourangeau et al., 2008).

## Study 1

### Methods

#### Participants

The required sample size was determined through a power analysis using G*Power. The results indicated that 256 participants (64 for each condition) were needed in order to detect medium effect sizes (*d* = 0.50) in a *t* test with an alpha of 0.05 and a power of 0.80.

The experiment was published in Prolific, where we recruited 256 individuals (128 women, mean age = 31.39 years, *SD* = 10.42, and 128 men, mean age = 33.70 years, *SD* = 10.51; one woman’s age was missing). Participants had to live in Spain and be fluent in Spanish to take part in the study.

#### Materials and procedure

We built the contingency problems using a medical scenario as a frame. Participants had to assess the ability of a substance to cure a headache based on the information from a fictitious clinical trial (i.e., the substance was the candidate cause while recovery from the headache was the outcome). Under this premise, four different conditions were created based on the information display of the contingency problem: trial-by-trial (*trial* condition), contingency table with numbers (*numbers* condition), contingency table with icons (*icons* condition), and frequency tree (*tree* condition). Specific instructions for each condition are available in Appendix A.

**Trial-by-trial (trial)**. Participants were given instructions for the task and asked not to write down any of the information displayed. At the beginning of each trial, participants were informed that one patient was suffering from a headache. On top of that, a message would also appear saying either *The patient took the substance* or *The patient did not take the substance*. Afterward, they had to report whether they believed the headache would disappear, or not, by pressing a *yes* or *no* button, respectively. Next, a message would appear on the computer screen indicating whether the patient had, or had not, recovered from the headache after two hours. After that, a new trial would begin.

**Contingency table with numbers (numbers)**. Participants were provided with a 2 × 2 contingency table (see Fig. 2A) with some instructions so that they would be able to interpret it properly. In the different cells (*A*, *B*, *C*, *D*), it was written with figures the amount of (*A*) patients who had taken the substance and recovered, (*B*) patients who had taken the substance and still had a headache, (*C*) patients who had not taken the substance and did recover, and (*D*) patients who had not taken the substance and did not recover.

**Contingency table with icons (icons)**. The instructions and display structure were identical to the *numbers* condition. The only thing that varied was how the information was shown inside the cells. Instead of having figures, the data were represented using icons of faces. Icons representing patients who had taken the substance were orange, whereas the icons of those who had not taken it were blue. Moreover, the faces were either happy, if they had recovered from the headaches, or sad, if they had not (see Fig. 2B).

**Frequency tree (tree)**. Participants were presented with a frequency tree (see Fig. 2C) along with some text explanation, in order to understand the information given. In the boxes of the tree, there was information regarding the total number of patients in the clinical trial, the number of patients who took the substance, the number of patients who did not take it, as well as how many of them recovered and how many did not, both among those taking the substance and among those not taking it.

Participants were randomly assigned to one of the conditions. After visualizing the information, they had to rate, on a scale from 0 to 100, to what extent they thought that the substance impacted the patients’ recovery (effectiveness question; i.e., *To what extent do you think that the substance is effective against the headache?*). Numbers close to zero indicated that the substance was not effective for curing headaches, whereas values close to 100 indicated that the substance was completely effective (the visible labels of the scale were *not effective at all* for the minimum zero value, *moderately effective* for the medium value of 50, and *totally effective* for the maximum 100 value, and participants were instructed that they could enter any value between 0 and 100).

Previous studies have reported that causal illusions tend to be stronger when the probability of the candidate cause and the outcome are high (Blanco et al., 2013). For this reason, for all conditions, the values of the cells that we chose were 18, 6, 6, and 2 for cells A, B, C, and D, respectively, generating high probabilities of the outcome [$P\left(Outcome\right)=\frac{A + C}{A + B + C + D}=.75$] and the cause [$P\left(Cause\right)= \frac{A + B }{A + B +C + D}=.75$]. We expected that those values would generate strong causal illusions and, therefore, we would have a higher room to detect any decrease in the strength of causal illusions due to the impact of presentation formats. It must be noted that the task was not contingent ($\Delta P=0$) given that P(Outcome|Cause) and P(Outcome|¬Cause) were in both cases 0.75, meaning that the substance was not efficacious [$P\left(Outcome|Cause\right)= \frac{18}{18 + 6 }$ and $P\left(Outcome|\neg Cause\right)= \frac{6}{6 + 2}$].

### Results

We used R language under RStudio (Version 2023.03.1 + 446) software to conduct the statistical analysis. Descriptive analyses were computed using the package *doBy* (Højsgaard & Halekoh, 2022). In addition, we used the packages *dplyr* (Wickham et al., 2023), *FSA* (Ogle et al., 2023), and *diptest* (Maechler, 2023) during the inferential analyses. As for the plots, they were created using the *ggplot2* package (Wickham, 2016). Additionally, we also used JASP (Version 0.17.2.1) software to calculate the effect sizes and the Bayes factors (*BF*). Obtained values were interpreted according to the criterion presented in Wagenmakers et al.’s (2018) report, where values above 1, 3, and 10 represent, respectively, anecdotal, moderate, and strong evidence favoring the alternative (*BF*_10_) or null (*BF*_01_) hypothesis.

**Fig. 3:**
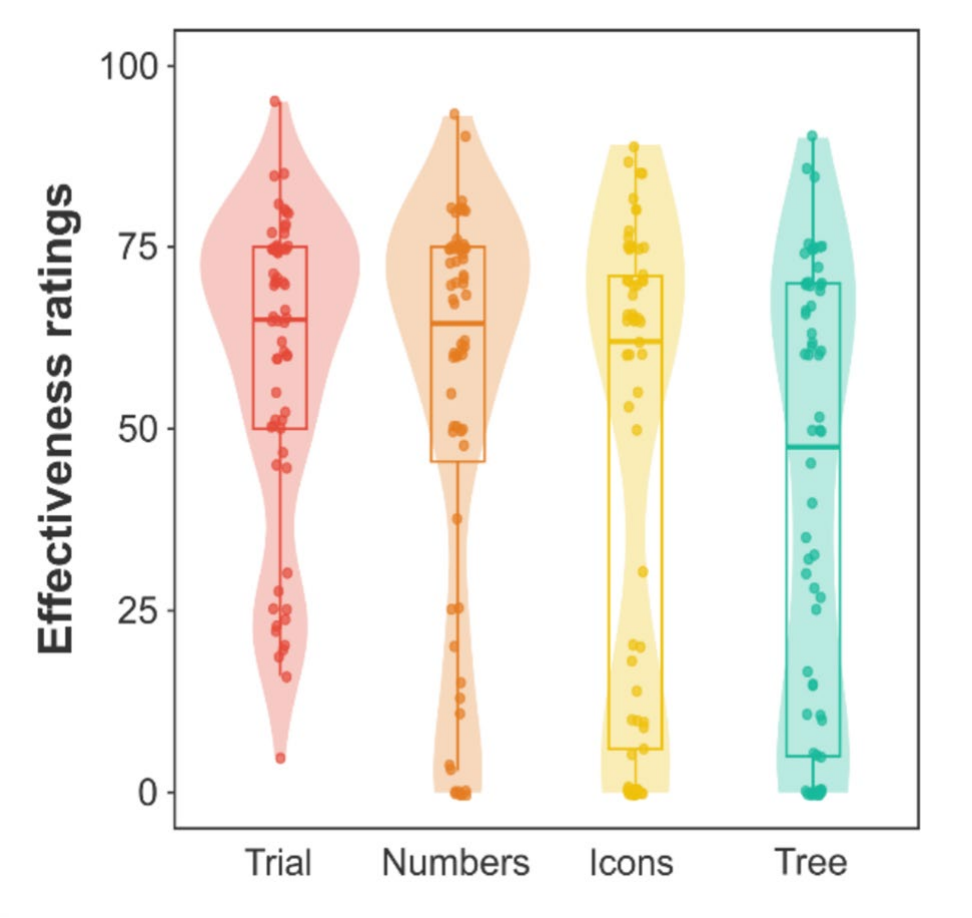
Effectiveness ratings observed in the trial, icons, numbers, and tree conditions in Study 1. *Note.* The distribution of effectiveness ratings of each condition is illustrated utilizing a violin and a box plot. For the box plot, the box limits define the interquartile range, showing the location of 50% of the data. The median is represented by a thick horizontal line allocated inside the mentioned box. The vertical lines depict the whiskers of the box plot. Additionally, individual effectiveness ratings are symbolized by dots. (Color figure online)

The mean effectiveness ratings for the *trial*, *numbers*, *icons*, and *tree* conditions were 59.02 (*SD* = *21.29*), 54.23 (*SD* = *28.19*), 44.62 (*SD* = *33.05*), and 40.05 (*SD* = *30.67*), respectively. Therefore, on average, it seemed that participants had developed a causal illusion to some extent. However, an inspection of the effectiveness rating distribution provided a sounder view. As shown in Fig. 3, although many participants developed strong causal illusions, some of them were able to detect there was no contingency between the variables. These two clusters seemed particularly visible in the *icons* and *tree* conditions, and suggested that the distribution of effectiveness ratings might not be unimodal. For this reason, we decided to conduct an additional analysis regarding the distribution of the data in each condition. Hartigan's dip test on the effectiveness ratings yielded significant results for the *icons*, *d* = 0.12, *p* < 0.001, and *tree* conditions, *d* = 0.10, *p* < 0.001, suggesting an absence of unimodality. In contrast, we found a non-significant deviation for the *trial* group, *d* = 0.05, *p* = 0.420, and only a marginally significant deviation for the *table* group,* d* = 0.06, *p* = 0.078.

As for the main analysis of the study, there were no differences in the effectiveness ratings between the *trial* and *numbers* condition*, t*(125) =  − 1.08*, p* = *0.2*83, *BF*_01_ = 3.12, *d* = − 0.19. Nevertheless, we found that the *trial* condition significantly differed from the icons, *t*(109) =  − 2.94, *p* = 0.004, *BF*_10_ = 8.40, *d* = − 0.52, and *tree* condition*, t*(112) =  − 4.05, *p* < 0.001, *BF*_10_ = 233.60, *d* = − 0.72.

Additionally, since the Shapiro–Wilk tests showed that effectiveness ratings did not follow a normal distribution in any of the conditions, minimum *W* = *0.81, p* < 0.001, we also decided to compare effectiveness ratings in the three summarized formats (*numbers*, *icons*, and *tree*) with those in the *trial* condition by means of Mann–Whitney *U* tests. Effectiveness ratings did not differ between the *trial* and *numbers* conditions, *U* = 1896.00, *p* = 0.563, *BF*_01_ = 3.48, *r*_s_ = − 0.06, while they were higher in the *trial* than in both the *icons*, *U* = 1588.00, *p* = 0.028, *BF*_10_ = 2.55, *r*_s_ = − 0.22, and the *tree* conditions, *U* = 1268.00, *p* < 0.001, *BF*_10_ = 65.92, *r*_*s*_ = − 0.37.

We also tested whether all summarized formats were equally effective in reducing the causal illusions. The one-way analysis of variance (ANOVA) indicated significant differences in the effectiveness ratings between the different summarized groups, *F*(2,190) = 3.56, *p* = 0.030, *η*^*2*^ = 0.04. Post hoc analyses using a Bonferroni correction showed there were only differences between the *numbers* and *tree* condition, *t*(190) = 2.61, *p*_adj_ = 0.029, *d* = 0.46, while the effectiveness ratings of the *icons* condition did not differ either from the *numbers*, *t*(190) =  − 1.78, *p*_adj_ = 0.231, *d* = − 0.31, or *tree* conditions, *t*(190) = 0.85, *p*_adj_ = 1.000, *d* = 0.15. In addition, given the violations of normality assumptions, we conducted the analogous nonparametric test. Consistent with the previous analysis, the Kruskal–Wallis test revealed that there were statistical differences between the different summarized groups, *H*(2) = 7.23, *p* = 0.027, *η*^*2*^ = 0.02. Post hoc analyses found statistical differences between the *tree* and *numbers* condition, *Z* = 2.68, *p*_adj_ = 0.022, *r*_s_ = 0.28 (i.e., effectiveness ratings were significantly greater for the *numbers* than for the *tree* visualization). The *icons* condition did not differ either from the *numbers*, *Z* = − 1.57,* p*_adj_ = 0.350, *r*_s_ = 0.15, or from the *tree* condition, *Z* = 1.12, *p*_adj_ = 0.790, *r*_s_ = 0.10.

### Discussion

In Study 1, we explored how trial-by-trial and summarized displays can have a differential impact on effectiveness judgments. The results suggest that frequency trees and contingency tables with icons might help people detect that there is no relation between two variables under null contingency circumstances. More specifically, frequency trees were the presentation format that best reduced causal illusions. A possible reason for this result is that they provided information regarding the total number of patients, the number of those who took the substance, and the number of those who did not take it (i.e., 32, 24, and 8, respectively, in Fig. 2C). Although these values can be easily computed based on the frequencies provided in the contingency table, only the frequency tree explicitly shows them. Note that these values are necessary for a proper assessment of contingency. Bearing in mind the ΔP formula (see Formula 2), the number of patients who took the substance corresponds with the denominator of the first fraction in the formula (i.e., A + B), while the total number of patients who did not take it coincides with the denominator of the second division (i.e., C + D). Although people usually neglect these values (i.e., denominator neglect bias), they are essential for a proper assessment of contingency, and enhancing their detection can improve it (Cooper & Vallée-Tourangeau, 2021). Therefore, one possible explanation is that frequency trees, by explicitly depicting these values, make the denominator more salient, which in turn enhances the assessment of contingency.

It is worth noting that the performance under the *icons* condition was somewhat in the middle of that observed in the *numbers* and *tree* conditions. Following the idea that trees might promote better causal assessment due to their explicit presentation of the denominators to be taken into account, it might be possible that our *icons* condition benefited from the colors we used. As shown in Fig. 2B, all patients taking the substance were presented in orange, and all patients not taking it were presented in blue. We hypothesized that colors may have facilitated grouping the information into two separate units: patients who took the substance and patients who did not (i.e., Cells A and B vs. Cells C and D), thereby, making the denominators more noticeable and easily accessible. Simultaneously, this differentiation may have also stressed the importance of comparing the information between these two groups, resulting in a better contingency assessment.

Therefore, our goal in Study 2 was to assess whether causal reasoning would be enhanced if the marginal frequencies were made explicitly available in a condition involving contingency tables with numbers. Furthermore, we also wanted to test whether using colors to guide the calculation of the denominators would help detect the lack of contingency. For this purpose, we compared three different visualizations of contingency tables with numbers: standard contingency tables showing only conditional frequencies (*control* condition), contingency tables showing both conditional and marginal frequencies (*marginal* condition), and contingency tables only involving conditional frequencies but with those frequencies color coded (*color* condition). In addition, for exploratory purposes, we recorded participants’ reasoning strategies (through an open question; see Methods) in order to deepen into the multimodal distribution of effectiveness judgments observed in Study 1.

## Study 2

### Methods

#### Participants

We recruited 390 participants via Prolific (195 women, mean age = 32.27 years, *SD* = 10.90, and 195 men, mean age 34.80 years, *SD* = 11.20, the age of one man was missing), which is sufficient to detect low-to-medium effect sizes (*d* = 0.35) in a *t* test (power = 0.80, alpha = 0.05). To participate in the study, subjects had to live in Spain and be fluent in Spanish.

#### Materials and procedure

We used the same task designed for Study 1. There were three conditions based on the display in which the information was presented, and participants were randomly assigned to one of them. The instructions for each of the conditions can be found in Appendix B.

First, in the *control* condition, participants were provided with a 2 × 2 contingency table along with an explanation to understand the table. The cells contained the frequencies conditioned on the substance intake and patients’ recovery. This condition was identical to that employed in Study 1 (*numbers* condition; *see* Fig. 2A).

**Fig. 4:**
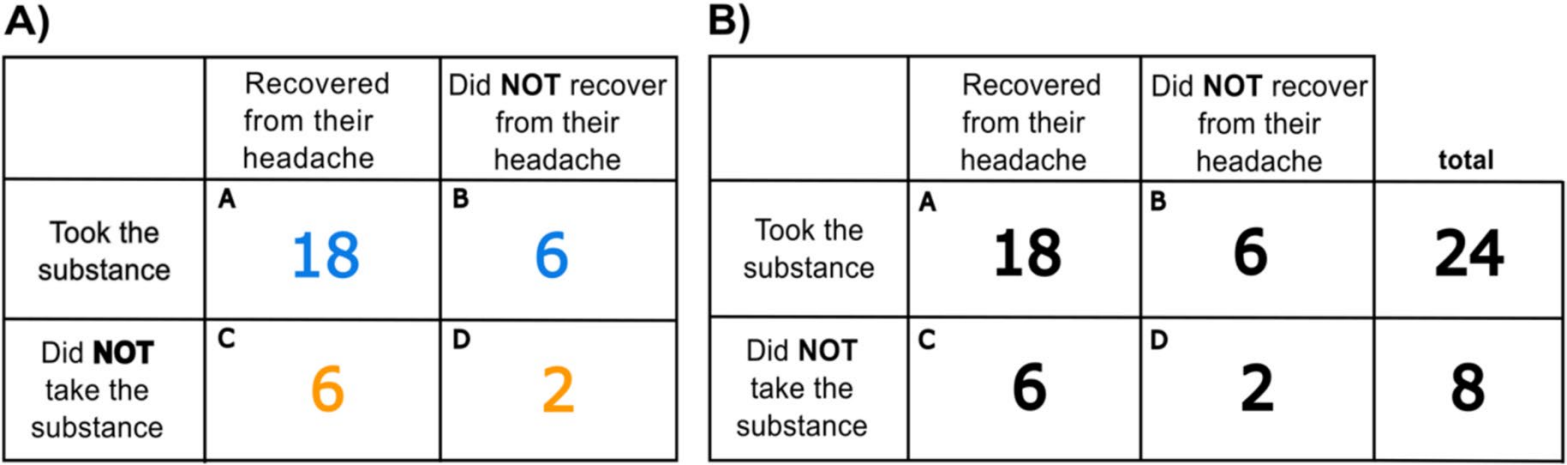
The information visualization formats employed in the color **A) **and marginal **B)** conditions in Study 2. (Color figure online)

Second, in the *color* condition, participants were given an equal contingency table and instructions as that of the *control* condition, except that now the frequencies from each table row were differentially colored (see Fig. 4A). Specifically, the cells corresponding to cases in which the substance was taken were blue, and those referring to cases in which the substance was not taken were orange.

Lastly, for the *marginal* condition, participants were provided with a 2 × 3 table (see Fig. 4B) and instructions. Along with the four cells showing how many patients did and did not recover, both among those taking and not taking the substance, two additional cells represented the total number of patients who took the substance and the total number of patients who did not take it. Thus, not only conditional frequencies but also key marginal ones were explicitly available.

After visualizing the corresponding table, participants from all three conditions responded to the effectiveness question (see Study 1). Additionally, as an exploratory measure, we recorded the participants’ reported reasoning strategy on an open-ended question (for a similar approach, see Batanero et al., 2015; Mata et al., 2015). For this purpose, after they gave the effectiveness rating, we asked participants to write down the information they had taken into account and the reasoning they had followed to reach their conclusion (i.e., *We asked you to indicate on a scale from 0 to 100 the extent to which you believed the substance was effective against headaches and you indicated a value of [—]. Given your previous answer, could you explain what information you took into account and what reasoning you followed to reach that conclusion?*).

### Results

We used JASP (Version 0.18.3) to analyze the data and to compute the Bayes factors for the tests performed. Plots were generated using *gglot2* package (Wickham, 2016) in RStudio (Version 2023.12.1 + 402).

**Fig. 5:**
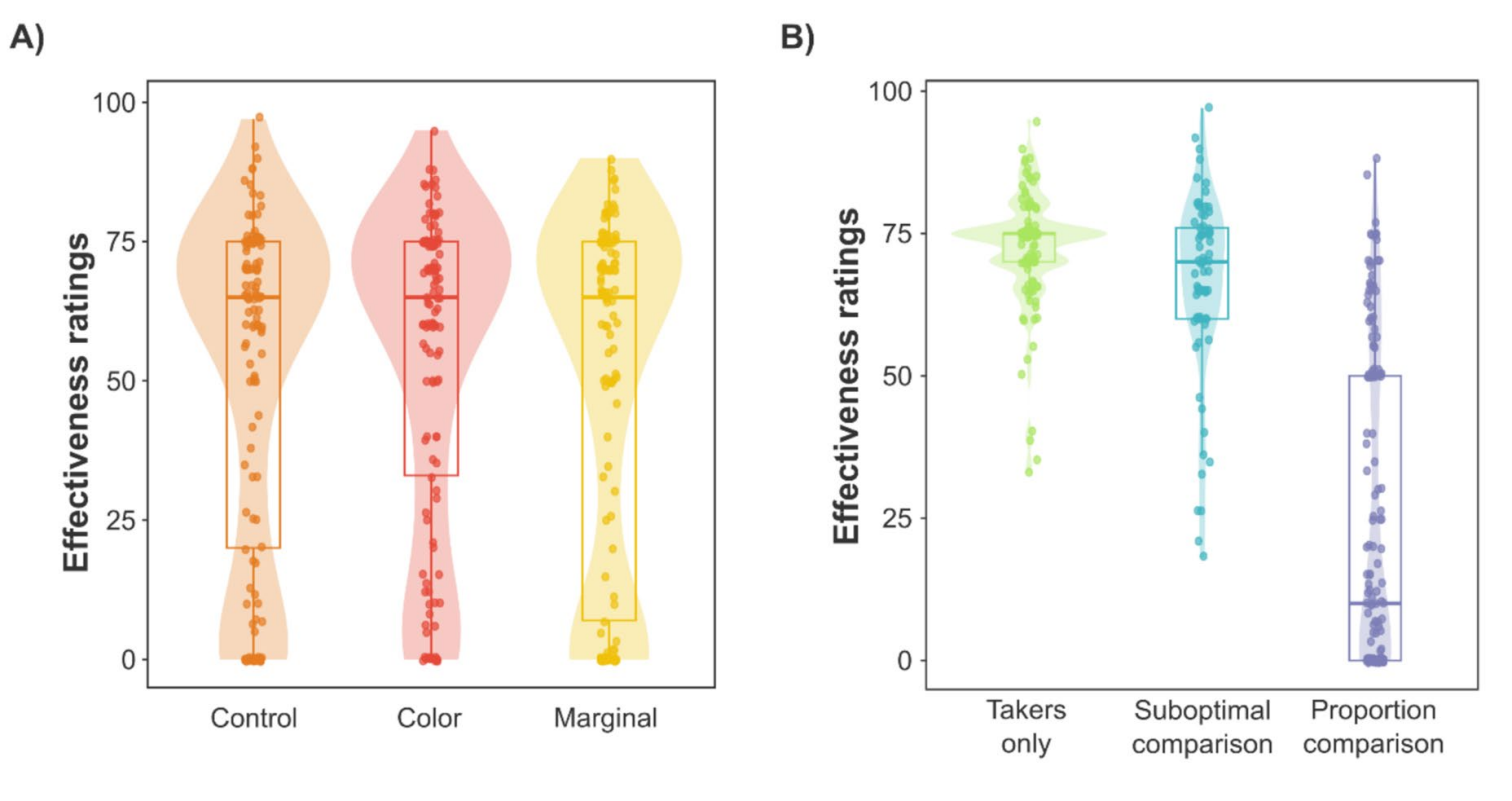
Effectiveness ratings observed according to the presentation format **A)** and the participant’s reported reasoning strategy **B) **in Study 2. *Note.* The distribution of the effectiveness ratings of each presentation format **A)** and each reasoning strategy **B)** are illustrated with a violin and a box plot. For the box plot, the box limits define the interquartile range, showing the location of 50% of the data. The median is represented by a thick horizontal line allocated inside the box and the vertical lines depict the whiskers of the box plot. Additionally, individuals’ effectiveness ratings are symbolized by dots. (Color figure online)

Mean effectiveness ratings for the *control*, *color,* and *marginal* conditions were 50.61 (*SD* = 30.22), 53.26 (*SD* = 28.45), and 48.51 (*SD* = 31.84), respectively. The *control* condition did not differ from the *color*, *t*(259) =  − 0.73, *p* = 0.468, *BF*_01_ = 5.73, *d* = − 0.09, or from the *marginal* condition, *t*(259) = 0.55, *p* = 0.585, *BF*_01_ = 6.39, *d* = − 0.07 (see Fig. 5A). Moreover, given that data violated the normality assumption, minimum *W* = 0.81, *p* < 0.001, we conducted an analogous nonparametric test. We also found that there were no differences in the effectiveness ratings either between the *control* and *color* conditions, *U* = 8084.50, *p* = 0.480, *BF*_01_ = 5.66, *d* = − 0.05, or between the *control* and *marginal* conditions, *U* = 8627.00, *p* = 0.853, *BF*_01_ = 6.91, *d* = 0.01.

More interesting results emerged in relation to our exploratory open-ended question. To analyze the participants'reasoning strategy, we first conducted a preliminary revision of all the participants’ responses, which led us to define the different categorization levels. Answers were categorized based on the strategy complexity level. We coded the participant strategy as *Takers only* if they had only considered the frequencies regarding the patients who had taken the substance (i.e., Cell A and/or Cell B). In contrast, a strategy was categorized as *Suboptimal comparison* if the participant had to some extent considered the data regarding both the patients who took the substance and those who did not, but they had not compared these groups of patients in terms of ratios or proportions of recovery (e.g., by comparing the frequency in Cell A with that in C, or by comparing the frequency in Cell A with the sum of frequencies of the rest of the cells, among other suboptimal alternatives). Lastly, those participants who took into consideration both groups of patients and compared them in terms of ratios or proportions of recovery (i.e., A and B vs. C and D) were coded as *Proportion comparison*. Responses were first categorized by two of the authors (Barreiro and del Carpio). Afterward, the other two authors (Barberia and Rodríguez-Ferreiro) revised those answers for which there was no agreement in the previous classification. The answers of 46 participants were not included in the analysis, as they did not fit into any of these categories. Examples of answers disregarded were those stating that the participant had based their reasoning on the information given but without mentioning the specific cells used, not basing their judgments on any information proportioned, or not answering the question.

We obtained an adequate interrater agreement on the participant’s strategy (*k* = 0.80). The frequencies of *takers only*, *suboptimal comparison,* and *proportion comparison* strategies were 117 (34.01%), 72 (20.93%), and 155 (45.06%), respectively. Further examination of this exploratory measure revealed that the reported reasoning strategy was associated with the strength of the causal illusion observed in effectiveness ratings, *F*(2, 341) = 203.08, *p* < 0.001,* η*^*2*^ = 0.54. As shown in Fig. 5B, the strongest causal illusions appeared to emerge in the subjects reporting the *takers only* strategy (*M* = 72.34, *SD* = 9.99), followed by those performing a *suboptimal comparison* (*M* = 66.13, *SD* = 16.48), and those falling in a *proportion comparisons* category (*M* = 24.41, *SD* = 27.80). Post hoc comparisons using Holm’s correction confirmed these impressions. Effectiveness ratings were higher for those participants employing the *takers only* strategy, compared with both those categorized as *suboptimal comparison* users and as *proportion comparison* users, *t*(341) = 1.98, *p*_*adj*_ = 0.049, *d* = 0.30, and *t*(341) = 18.67, *p*_*adj*_ < 0.001, *d* = 2.28, respectively. Moreover, *suboptimal comparison* users showed higher effectiveness ratings than *proportion comparison* users,* t*(341) = 13.95, *p*_*adj*_ < 0.001 *d* = 1.99. Since the data violated the homoscedasticity assumptions, *F*(2, 341) = 105.75, *p* < 0.001, we conducted nonparametric analogous tests, which led to the same conclusions. Accordingly, effectiveness ratings differed based on the strategy participants used, *H*(2) = 179.57, *p* < 0.001,* η*^*2*^ = 0.52. We also found that the effectiveness ratings of the *takers only* significantly differed from those participants employing *the suboptimal comparison*, *Z* = 1.97, *p*_adj_ = 0.049, *r*_s_ = 0.23, and the *proportion comparison*, *Z* = 12.65, *p*_adj_ < 0.001, *r*_s_ = 0.87. The *suboptimal comparison* and *the proportion comparison* groups also differed in their effectiveness ratings, *Z* = *8.79, p*_adj_ < 0.001, *r*_s_ = 0.77.

It is worth noting that a substantial part of the participants (39.35%) who used the *proportion comparison* strategy gave ratings of zero. Nevertheless, a portion of these subjects still believed that the pill was effective. The inspection of their open responses indicated there were different reasons for this outcome. For instance, some participants were able to detect that the proportions of recoveries were equal with and without the substance, but still decided to provide a high causal rating. Their explanations suggested in some cases that they believed they had to indicate the P(Outcome|Cause) instead of the contingency level. In other cases, they emphasized that either the small sample size of the study or the differences in sample size between patients taking and not taking the substance were problematic. We also encountered a few participants who did not correctly calculate the proportion of recoveries for patients who took or did not take the substance.

Finally, as an additional exploratory analysis suggested by a reviewer, we examined the impact of the presentation format on the reasoning strategy and found no significant effect, χ^2^(4, 344) = 2.79, *p* = 0.594, Cramer’s* V* = 0.06, *BF*_01_ = 228.04.

### Discussion

In Study 2, we compared how different layouts of contingency tables with numbers influence effectiveness judgments. Contrary to our hypotheses, our results indicated there was no benefit in including marginal frequencies or in using colors to highlight the reference groups that needed to be compared.

In addition, our exploratory analysis revealed that the complexity of the reasoning strategy was associated with the accuracy of the effectiveness judgments, as participants using the *proportion comparison* strategy developed the weakest causal illusions on average. This strategy yields a calculation similar to that of the ΔP rule. As a result, participants might have been more likely to detect the absence of contingency.

One of the main focuses of Study 2 was to test the hypothesis that providing the key marginal frequencies could benefit the assessment of contingency, since we had presumed this feature yielded the difference found between frequency trees and contingency tables in Study 1. Nonetheless, Study 2 suggested that explicitly depicting these frequencies was not necessarily helpful. Consequently, we speculated that trees’ efficacy might not be attributed simply to their explicit presentation of key marginal frequencies, but to their hierarchical structure. More precisely, we presumed that the branches of the tree might pave the way for making the computation of ΔP more accessible. As a matter of fact, in Bayesian reasoning, it has been proposed that the efficacy of frequency trees lies in their nested structure. The design of these trees facilitates the comprehension of the subset's relations, which produces representational and computational benefits (Böcherer-Linder & Eichler, 2017; Yamagishi, 2003).

Accordingly, the goal of Study 3 was to examine whether the benefits of using a frequency tree compared with a contingency table with numbers observed in Study 1 could be explained by a stronger tendency to use ΔP rule-like strategies (i.e., *proportion comparison*) in the former than in the latter. Moreover, we expected frequency trees to generate weaker causal illusions than contingency tables (Hypothesis 1, replicating Study 1) and the strength of that illusion to be dependent on the reported reasoning strategy (Hypothesis 2, replicating Study 2). Crucially, we presumed there would be an association between the information layout and the reasoning strategy employed (Hypothesis 3). More precisely, we hypothesized that individuals would be more likely to use a ΔP rule-like strategy when representing the data in a frequency tree than when depicting it in a contingency table.

## Study 3

### Methods

#### Participants

We estimated the required sample size to detect a medium effect size in an ANOVA, *f* = 0.25, power = 0.95, alpha = 0.05 through a power analysis in G*Power. As a result, we recruited 251 participants in Prolific. All of them were UK citizens, and their first language was English. Furthermore, the sample was composed of 126 women, mean age = 41.41 years, *SD* = 14.28, and 125 men, mean age = 40.88 years, *SD* = 13.92.

#### Materials and procedure

We used the same task as in the previous studies. Note that the contingency problems were identical to the *tree* and *numbers* conditions in Study 1, and that participants were randomly assigned to one of the conditions. Furthermore, since participants were English speakers, materials were translated into English and revised by a native speaker (see Appendix C). As in Study 2, after visualizing the data, subjects first emitted an effectiveness rating and then answered an open question regarding their reasoning strategy.

### Results

Data were analyzed on RStudio (Version 2023.12.1 + 402) using the packages *FSA* (Ogle et al., 2023)*,* and *doBy* (Højsgaard & Halekoh, 2022). In addition, Bayes factors and effect sizes were computed in JASP (Version 0.18.3).

**Fig. 6:**
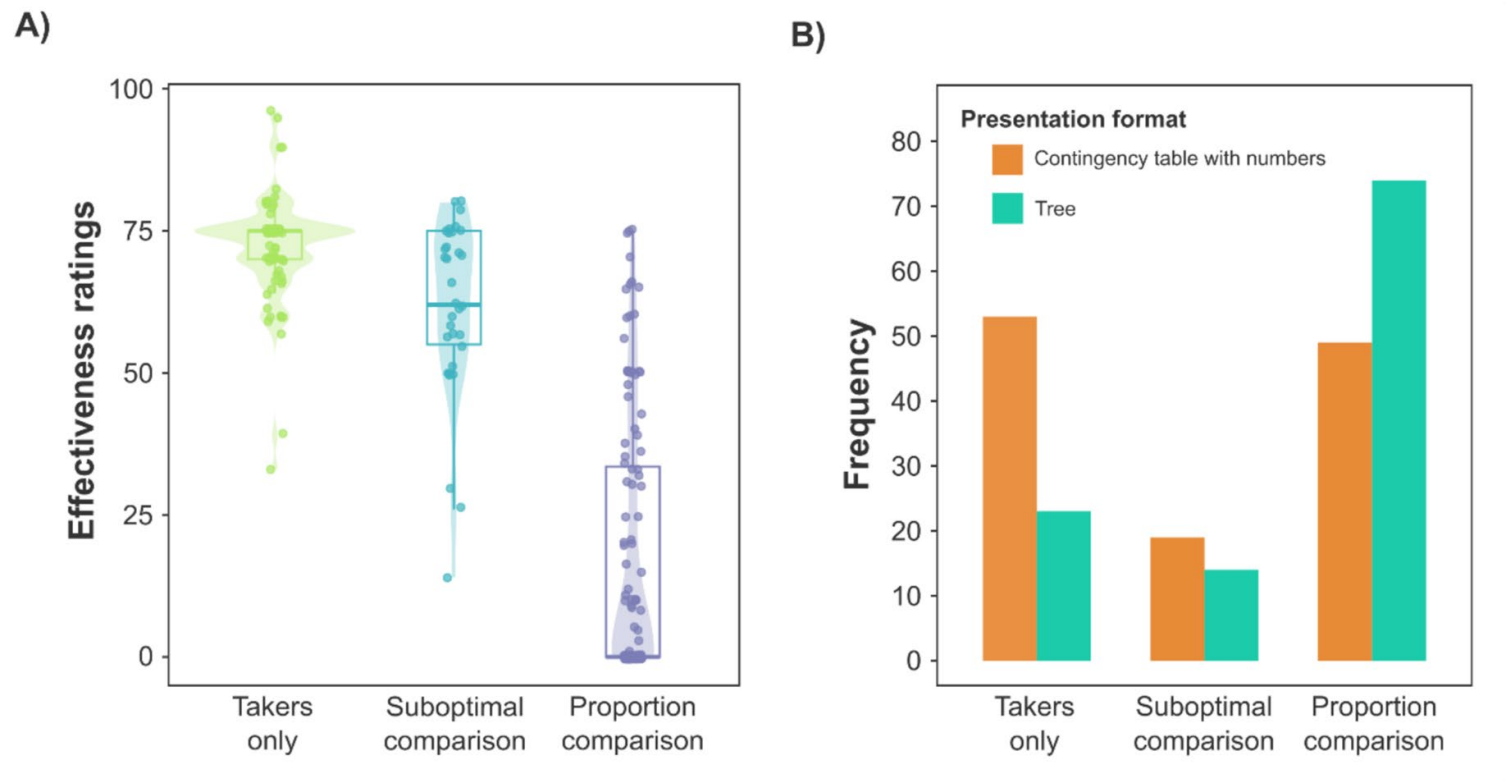
Impact of reasoning strategies on effectiveness judgments **A)** and the distribution of reasoning strategies in each condition **B)** in Study 3. *Note.* **A)** Effectiveness judgments emitted based on the reasoning strategies are depicted in a box plot. The interquartile range is defined by the box limits and the median by a thick horizontal line allocated inside the box. The vertical lines represent the whiskers of the boxplot. Additionally, the distributions of the effectiveness ratings are also illustrated in a violin plot and individuals’ effectiveness ratings are symbolized with dots. **B)** Frequency of participants reporting each reasoning strategy in each of the presentation formats. (Color figure online)

As for the examination of the open-ended questions (see Fig. 6A), we followed the same procedure described in Study 2 to categorize participants'responses. We excluded 19 answers that did not meet the criteria to be included in any of the three categories. The frequency of each reasoning strategy was 76 (32.76%), 33 (14.22%), and 123 (53.02%) for the *takers only*, *suboptimal comparison,* and *proportion comparison*, respectively. The interrater agreement on the participant’s strategy was *k* = 0.81. Our preregistered 2 (presentation format) × 3 (reasoning strategy) ANOVA on effectiveness ratings showed a significant effect of the reasoning strategy, *F*(2, 226) = 201.68, *p* < 0.001,* η*^*2*^ = 0.63, while both the main effect of the presentation format, *F*(1, 226) = 2.35, *p* = 0.127, *η*^*2*^ = 0.00, and the interaction, *F*(2, 226) = 1.77, *p* = 0.173,* η*^*2*^ = 0.00, did not reach significance. However, due to the violations of the homoscedasticity assumptions, *F*(5, 226) = 18.64, *p* < 0.001, we also conducted non-parametric tests on our data, which led to different conclusions.

First, regarding the influence of presentation format on effectiveness ratings (Hypothesis 1), as expected, but contrary to the conclusions drawn from the ANOVA, the Mann–Whitney *U* test indicated that ratings were higher when being exposed to contingency tables with numbers (*n* = 125, *M* = 50.21, *SD* = 30.17) than to frequency trees (*n* = 126, *M* = 35.89, *SD* = 31.96), *U* = 9971.50, *p* < 0.001, *BF*_10_ = 49.17, *r*_*s*_ = 0.27.

Second, regarding the impact of the reasoning strategy on effectiveness ratings (Hypothesis 2), and consistent with the results of our previous ANOVA, a Kruskal–Wallis analysis revealed a statistically significant effect of the reasoning strategy on effectiveness ratings, *H*(2) = 151.84, *p* < 0.001, *η*^*2*^ = 0.65. As shown in Fig. 6A, participants who reported using the *proportion comparison* strategy seemed to develop the weakest causal illusions (*M* = 17.20, *SD* = 23.32), followed by those performing a *suboptimal comparison* (*M* = 61.55, *SD* = 17.77), and those considering *takers only* (*M* = 72.18, *SD* = 9.41). For further examination, we performed a Dunn test applying Holm correction. We found statistically significant differences in effectiveness ratings among all three reasoning strategies: *takers only* and *suboptimal comparison* (*Z* = 2.07, *p*_adj_ = 0.038, *r*_s_ = 0.42), *takers only* and *proportion comparison* (*Z* = 11.85,* p*_adj_ < 0.001, *r*_s_ = 0.94), and *suboptimal comparison* and *proportion comparison* (*Z* = 6.62, *p*_adj_ < 0.001, *r*_s_ = 0.84).

Lastly (Hypothesis 3), we examined the association between the format of presentation and the strategy employed. As illustrated in Fig. 6B, most of the participants in the *tree* condition used the *proportion comparison* strategy. In contrast, in the *contingency table with numbers* condition, the participants were more evenly distributed among the *takers only* and *proportion comparison* strategy. Consistent with these observations, we found a significant association between the format of presentation of the problem and the type of reasoning strategy reported, X^2^(2, 232) = 17.28, *p* < 0.001, Cramer’s* V* = 0.27, *BF*_10_ = 198.40.

## General discussion

Much of the recent literature on causal illusions has focused on studying this phenomenon in a trial-by-trial presentation (Barberia et al., 2019; Blanco et al., 2018; Chow et al., 2019; Griffiths et al., 2018; Moreno-Fernández et al., 2017; Torres et al., 2020). Our research focused on investigating whether different summarized displays of analogous information would reduce causal illusions and inquiring into the features that contributed to this decrease.

Study 1 disclosed that contingency tables with icons and frequency trees induced weaker causal illusions than a trial-by-trial presentation, while there was no benefit over trial-by-trial condition when the information was presented in a contingency table with numbers. Study 2 indicated that neither including marginal frequencies nor introducing colors to emphasize the groups of data to be compared produced a decrease in causal illusions when being exposed to contingency tables with numbers. Moreover, this second study indicated an association between participants’ reported reasoning strategy and their effectiveness ratings. Specifically, those participants who reported ΔP rule-like mental operations (i.e., comparing ratios or proportions of recovery between patients who took and did not take the drug) provided effectiveness ratings that were closer to zero, indicating that they had developed less intense causal illusions. Finally, Study 3 replicated the advantage of trees over contingency tables with numbers (as shown by the results of the nonparametric test), the association between reasoning strategies and causal illusions, and, importantly, it demonstrated that ΔP rule-like mental operations were more likely in the former than in the latter format of presentation.

The observation that the display of information in different summarized formats influences how participants solve the task extends findings from previous studies. With regard to this, Arkes and Harkness (1983) evaluated the reasoning strategies elicited by trial-by-trial and contingency tables. They found that when they employed contingency tables to present the information, the sum of the diagonals (which, in our data-driven categorization, would map onto our *suboptimal comparison* strategy) and the ΔP rule (which would map onto our *proportion comparison* strategy) were the most frequently used strategies. In contrast, for the trial-by-trial presentation, the Cell A and A–B strategies (which would both map onto our *takers only* strategy) were the predominant ones. Similarly, Ward and Jenkins (1965) found that when presenting the data in a contingency table, participants were more likely to use the ΔP rule compared with when a trial-by-trial presentation was used. We did not record the reasoning strategy for the trial-by-trial condition, so our results and those of these studies cannot be directly compared. Nevertheless, note that the lack of differences between the intensity of causal illusions corresponding to trial-by-trial and contingency tables with numbers that we observed in Study 1 might go against the use of differential strategies by the participants, so further research is needed to disentangle the reasons for these potentially conflicting results.

Among the different summarized formats tested, our research indicates that frequency trees are the most effective visualization to reduce causal illusions. A possibility is that the benefit of frequency trees emerges from their hierarchical structure, where contingency information is placed on nodes. The total sample of patients is depicted at the highest node, while, at the lower levels, information is grouped based on whether or not the patient took the substance and whether or not they recovered. As the branches connecting these nodes explicitly depict how these are embedded, it might be easier to understand the importance of calculating the proportion of recoveries. Note that this is parallel to the observed benefits of trees for Bayesian reasoning. For instance, Bruckmaier et al. (2019) showed that trees aid in avoiding joint occurrence errors (calculating the joint probability between the two key variables, or, in the usual positive predictive value problem, dividing the amount of positive tests in patients with the disease by the total amount of patients). In our causal inference problems, the sequential structure of the tree might help in understanding the need to calculate a rate between the amount of patients taking the drug and recovering and the total amount of patients taking the drug [$\frac{A}{A + B}$], instead of focusing only on the first value (Cell A strategy) or just comparing it with the total amount of patients [$\frac{A}{ A + B + C + D}$]. Additionally, in our case, the nested-set structure of the tree might also help in understanding the need to compare the two relevant conditional probabilities by presenting them side by side in the two branches of the tree. Following Tubau et al. (2019), this might be accomplished by creating “a better alignment between presented and requested relationships” (p. 1808).

Overall, our research indicates that the format of presenting the information matters if we want to facilitate a correct estimation of contingency. Given the possible role of causal illusion as a facilitator of unwarranted beliefs (Torres et al., 2020, 2022), presenting information using frequency trees could also be helpful in debiasing interventions. In this regard, a recent study by Chow et al. (2024) also suggests that providing instructions that emphasize the relevance of base rates of recovery (i.e., the probability of getting cured given the cause and the probability of getting cured without the cause) helps reduce causal illusions when information is presented in a summarized format.

Still, it is important to notice that, in our experiment, participants lacked specific preconceptions about the substance’s effectiveness. However, as evidenced by Vicente et al. (2023) and Blanco et al. (2018), people tend to be more susceptible to developing causal illusions that fit their prior beliefs. These prior beliefs can prompt a reluctant attitude to change by paying attention to confirming information and disregarding contradicting evidence (Azzopardi, 2021; Knobloch-Westerwick et al., 2015). Therefore, it is plausible that people with prior expectations could be less persuaded by the effects of graphical visualizations, potentially constraining the observed effects.

Furthermore, it must be kept in mind that we measured the utility of the formats based on the assessment of a single problem. Consequently, it is difficult to ascertain to what extent the pattern observed would be replicated if frequencies in Cells A, B, C, or D changed. In this regard, studies on contingency tables suggest that the detection of a null contingency can be facilitated, or hindered, depending on the frequencies depicted in the cells (Kao & Wasserman, 1993). That is, when the four cell frequencies are different, it is harder to detect that there is no relation between the two variables compared with when there are pairs of identical cells (e.g., A = B ≠ C = D, A = C ≠ B = D) or all the cells are equal. Therefore, the pattern observed may be subjected to the difficulty level of the problem. Further research should examine this potential interaction by incorporating other cell frequencies into their tasks.

In addition, our investigation opens the door for new research lines in the field. As our results revealed, making nested-set relationships visible might promote correct causal inferences. Still, frequency trees are not the only summarized visualizations explicitly depicting these relations. Stacked bar graphs and unit squares can serve this purpose too. The latter has demonstrated promising results in Bayesian reasoning, surpassing even frequency trees (Böcherer-Linder & Eichler, 2017). Moreover, it should be noted that the current study used a specific structure that included the information regarding the presence or absence of the cause at the middle level of the tree and the information relating to the outcome at the lower level. In our view, presenting the data in this order is key to facilitate an adequate understanding of the structure of the information. Nevertheless, a tree can also be structured the other way around (see Vallée-Tourangeau et al., 2008), and further studies should be conducted to confirm or refute our intuition.

In summary, the current work provides encouraging results on the possible use of frequency trees to protect people against causal illusions. Although there is still work to be done, we hope this research can serve as a turning point in the development of interventions.

## Data Availability

The data for all experiments are available at OSF (https://osf.io/n3vu7/?view_only=d34cf1ad2e1543ef9645be2de2feaa28), and we preregistered Study 2 (https://aspredicted.org/CVF_34H) and Study 3 (https://aspredicted.org/1V9_LCM).

## Appendix A. Study 1: Original contingency problems instructions (in Spanish)

### Trial condition

Vas a participar en un experimento. Por favor concéntrate en la tarea hasta su finalización. No debes tomar notas de la información que te presentamos.

Imagina que eres un/a investigador/a que está explorando **hasta qué punto una sustancia experimental funciona como tratamiento contra el dolor de cabeza**.

A continuación vas a observar, una a una, **diversas fichas que describen los episodios de dolor de cabeza de diferentes pacientes que participaron en un ensayo clínico**. Te informaremos sobre si cada paciente ha tomado la sustancia durante el episodio o si, por el contrario, no ha tomado nada. Tú deberás indicar si piensas que el dolor de cabeza del paciente desaparecerá en las siguientes dos horas o no. A continuación, te diremos si el dolor de cabeza desapareció o no, y pasarás a observar la siguiente ficha.

Recuerda: debes intentar **averiguar hasta qué punto la sustancia experimental es efectiva**.

Cuando hayas observado varias fichas de pacientes te preguntaremos sobre la sustancia.

### Numbers condition

Vas a participar en un experimento. Por favor concéntrate en la tarea hasta su finalización. No debes tomar notas de la información que te presentamos.

Imagina que eres un/a investigador/a que está explorando **hasta qué punto unasustancia experimental funciona como tratamiento contra el dolor de cabeza**.

A continuación vas a observar **una tabla en la que se describen los episodios de dolor de cabeza de diferentes pacientes que participaron en un ensayo clínico**. Recuerda: debes intentar **averiguar hasta qué punto la sustancia experimental es efectiva**. En las distintas casillas (***A***, ***B***, ***C***, ***D***) encontrarás información sobre cuántos pacientes tomaron la sustancia durante el episodio y cuántos no la tomaron, y también información sobre en cuántos de estos pacientes el dolor de cabeza desapareció en las siguientes dos horas:

- ***A*** - Número de pacientes que tomaron la sustancia y en los que el dolor de cabeza desapareció.
- ***B*** - Número de pacientes que tomaron la sustancia y en los que el dolor de cabeza **NO** desapareció.
- ***C*** - Número de pacientes que **NO** tomaron la sustancia y en los que el dolor de cabeza desapareció.
- ***D*** - Número de pacientes que **NO** tomaron la sustancia y en los que el dolor de cabeza NO desapareció.

### Icons condition

Vas a participar en un experimento. Por favor concéntrate en la tarea hasta su finalización. No debes tomar notas de la información que te presentamos.

Imagina que eres un/a investigador/a que está explorando **hasta qué punto una sustancia experimental funciona como tratamiento contra el dolor de cabeza**.

A continuación vas a observar **una tabla en la que se describen los episodios de dolor de cabeza de diferentes pacientes que participaron en un ensayo clínico**. Recuerda: debes intentar averiguar **hasta qué punto la sustancia experimental es efectiva**. Dentro de las distintas casillas (***A***, ***B***, ***C***, ***D***) encontrarás información sobre cuántos pacientes tomaron la sustancia durante el episodio y cuántos no la tomaron, y también información sobre en cuántos de estos pacientes el dolor de cabeza desapareció en las siguientes dos horas. Esta información la verás reflejada través de iconos, cada icono representa un paciente.

- ***A*** - Número de pacientes que tomaron la sustancia y en los que el dolor de cabeza desapareció.
- ***B*** - Número de pacientes que tomaron la sustancia y en los que el dolor de cabeza **NO** desapareció.
- ***C*** - Número de pacientes que **NO** tomaron la sustancia y en los que el dolor de cabeza desapareció.
- ***D*** - Número de pacientes que **NO** tomaron la sustancia y en los que el dolor de cabeza **NO** desapareció.

### Tree condition

Vas a participar en un experimento. Por favor concéntrate en la tarea hasta su finalización. No debes tomar notas de la información que te presentamos.

Imagina que eres un/a investigador/a que está explorando **hasta qué punto una sustancia experimental funciona como tratamiento contra el dolor de cabeza**.

A continuación vas a observar **un diagrama en el que se describen los episodios de dolor de cabeza de diferentes pacientes que participaron en un ensayo clínico**. Recuerda: debes intentar **averiguar hasta qué punto la sustancia experimental es efectiva**. En las distintas casillas (***A***, ***B***, ***C***, ***D***, ***E***, ***F***, ***G***) encontrarás información sobre cuántos pacientes tomaron la sustancia durante el episodio y cuántos no la tomaron, y también información sobre en cuántos de estos pacientes el dolor de cabeza desapareció en las siguientes dos horas:

- ***A*** - Número total de pacientes.
- ***B*** - Número de pacientes que tomaron la sustancia.
- ***C*** - Número de pacientes que **NO** tomaron la sustancia.
- ***D*** - Número de pacientes que tomaron la sustancia y en los que el dolor de cabeza desapareció.
- ***E*** - Número de pacientes que tomaron la sustancia y en los que el dolor de cabeza **NO** desapareció.
- ***F*** - Número de pacientes que **NO** tomaron la sustancia y en los que el dolor de cabeza desapareció.
- ***G*** - Número de pacientes que **NO** tomaron la sustancia y en los que el dolor de cabeza **NO** desapareció.

## Appendix B. Study 2: Original contingency problems instructions (in Spanish)

### Conditional condition

Imagina que eres un/a investigador/a que está explorando **hasta qué punto una sustancia experimental funciona como tratamiento contra el dolor de cabeza**.

A continuación vas a observar **una tabla en la que se describen los episodios de dolor de cabeza de diferentes pacientes que participaron en un ensayo clínico**. Recuerda: debes intentar **averiguar hasta qué punto la sustancia experimental es efectiva**. En las distintas casillas (***A***, ***B***, ***C***, ***D***) encontrarás información sobre cuántos pacientes tomaron la sustancia durante el episodio y cuántos no la tomaron, y también información sobre en cuántos de estos pacientes el dolor de cabeza desapareció en las siguientes dos horas:

- ***A***—Número de pacientes que tomaron la sustancia y en los que el dolor de cabeza desapareció.
- ***B***—Número de pacientes que tomaron la sustancia y en los que el dolor de cabeza **NO** desapareció.
- ***C***—Número de pacientes que **NO** tomaron la sustancia y en los que el dolor de cabeza desapareció.
- ***D***—Número de pacientes que **NO** tomaron la sustancia y en los que el dolor de cabeza **NO** desapareció.

### Colored condition

Imagina que eres un/a investigador/a que está explorando **hasta qué punto una sustancia experimental funciona como tratamiento contra el dolor de cabeza**.

A continuación vas a observar **una tabla en la que se describen los episodios de dolor de cabeza de diferentes pacientes que participaron en un ensayo clínico**. Recuerda: debes intentar **averiguar hasta qué punto la sustancia experimental es efectiva**. En las distintas casillas (***A***, ***B***, ***C***, ***D***) encontrarás información sobre cuántos pacientes tomaron la sustancia durante el episodio y cuántos no la tomaron, y también información sobre en cuántos de estos pacientes el dolor de cabeza desapareció en las siguientes dos horas:

- ***A***—Número de pacientes que tomaron la sustancia y en los que el dolor de cabeza desapareció.
- ***B***—Número de pacientes que tomaron la sustancia y en los que el dolor de cabeza **NO** desapareció.
- ***C***—Número de pacientes que **NO** tomaron la sustancia y en los que el dolor de cabeza desapareció.
- ***D***—Número de pacientes que **NO** tomaron la sustancia y en los que el dolor de cabeza **NO** desapareció.

### Marginal condition

Imagina que eres un/a investigador/a que está explorando **hasta qué punto una sustancia experimental funciona como tratamiento contra el dolor de cabeza**.

A continuación vas a observar **una tabla en la que se describen los episodios de dolor de cabeza de diferentes pacientes que participaron en un ensayo clínico**. Recuerda: debes intentar **averiguar hasta qué punto la sustancia experimental es efectiva**. En las distintas casillas (***A***, ***B***, ***C***, ***D***, ***E***, ***F***) encontrarás información sobre cuántos pacientes tomaron la sustancia durante el episodio y cuántos no la tomaron, y también información sobre en cuántos de estos pacientes el dolor de cabeza desapareció en las siguientes dos horas:

- ***A***—Número de pacientes que tomaron la sustancia y en los que el dolor de cabeza desapareció.
- ***B***—Número de pacientes que tomaron la sustancia y en los que el dolor de cabeza **NO** desapareció.
- ***C***—Número total de pacientes que tomaron la sustancia.
- ***D***—Número de pacientes que **NO** tomaron la sustancia y en los que el dolor de cabeza desapareció.
- ***E***—Número de pacientes que **NO** tomaron la sustancia y en los que el dolor de cabeza **NO** desapareció.
- ***F***—Número total de pacientes que **NO** tomaron la sustancia.

## Appendix C. Study 3: Original contingency problems instructions (in English)

### Table condition

Imagine that you are a researcher who is exploring the **effectiveness of an experimental substance as a treatment for headaches**.

You will see **a table** below **describing the headache episodes of different patients who participated in a clinical trial**. Remember: Your goal is to **find out to what extent the experimental substance is effective**. In the different boxes (***A***, ***B***, ***C***, ***D***) you will find information about the number of patients who took the substance during the episode and the number of those who didn't, and also information on the number of patients who recovered from their headache within the next two hours:

- ***A*** - Number of patients who took the substance and recovered from their headache.
- ***B*** - Number of patients who took the substance and did **NOT** recover from their headache.
- ***C*** - Number of patients who did **NOT** take the substance and recovered from their headache.
- ***D*** - Number of patients who did **NOT** take the substance and did **NOT** recover from their headache.

### Tree condition

Imagine that you are a researcher who is exploring the **effectiveness of an experimental substance as a treatment for headaches**.

You will see **a diagram** below **describing the headache episodes of different patients who participated in a clinical trial**. Remember: Your goal is to **find out to what extent the experimental substance is effective**. In the different boxes (***A***, ***B***, ***C***, ***D***, ***E***, ***F***, ***G***) you will find information about the number of patients who took the substance during the episode and the number of those who didn't, and also information on the number of patients who recovered from their headache within the next two hours:

- ***A*** - Total number of patients.
- ***B*** - Number of patients who took the substance.
- ***C*** - Number of patients who did NOT take the substance.
- ***D*** - Number of patients who took the substance and recovered from their headache.
- ***E*** - Number of patients who took the substance and did NOT recover from their headache.
- ***F*** - Number of patients who did NOT take the substance and recovered from their headache.
- ***G*** - Number of patients who did NOT take the substance and did NOT recover from their headache.
